# Real-time gas evolution analysis of SEI formation in sodium-ion batteries using chip based electrochemistry mass spectrometry

**DOI:** 10.1039/d6fd00028b

**Published:** 2026-03-25

**Authors:** Shivam Sharma, Bethan J. V. Davies, Mary P. Ryan, Ifan E. L. Stephens, Maria Magdalena Titirici

**Affiliations:** a Department of Chemical Engineering, Imperial College London London SW7 2AZ UK m.titirici@imperial.ac.uk; b Department of Materials, Imperial College London London SW7 2AZ UK; c The Faraday Institution, Harwell Science and Innovation Campus Harwell OX11 0RA UK

## Abstract

The stability of the solid electrolyte interphase (SEI) in sodium-ion batteries remains a critical challenge for achieving long cycle lifetimes. In hard-carbon anodes, the interplay between microstructure, solvation chemistry, and electrolyte decomposition dictates SEI evolution, yet the mechanistic link between electrolyte decomposition, gas evolution, and interphase formation remains poorly resolved. Here, we apply chip-based electrochemistry-mass spectrometry (EC-MS) with picomole-per-second sensitivity to monitor real-time gas evolution during SEI formation on hard carbon in different solvent systems, representing one of the first applications of EC-MS to sodium-ion cells. Carbonate-based (EC:DMC) electrolytes exhibit pronounced gas evolution, dominated by C_2_H_4_ during the first discharge, consistent with reductive decomposition of EC and the formation of sodium ethylene dicarbonate and Na_2_CO_3_ rich interphase species. In contrast, ether-based electrolytes (diglyme) show strongly suppressed gas evolution, with only trace C_2_H_4_ detected, alongside improved rate capability and enhanced cycling stability over 100 cycles at 0.5 C. These differences are attributed to solvent-dependent solvation and interfacial kinetics, arising from the lower effective desolvation barrier and higher reductive stability of diglyme relative to carbonate solvents, leading to the formation of a thinner and more ion-permeable SEI. Collectively, these findings establish a direct connection between solvent chemistry, gas evolution, and SEI formation in sodium-ion systems and demonstrate the utility of *operando* chip-based EC-MS for resolving electrolyte degradation pathways.

## Abstract 2

*Operando* EC-MS reveals that EC:DMC produces C_2_H_4_ and CO during hard-carbon sodiation, indicating pronounced solvent decomposition. In contrast, diglyme suppresses gassing and promotes a thinner, more inorganic interphase.

## Graphical abstract

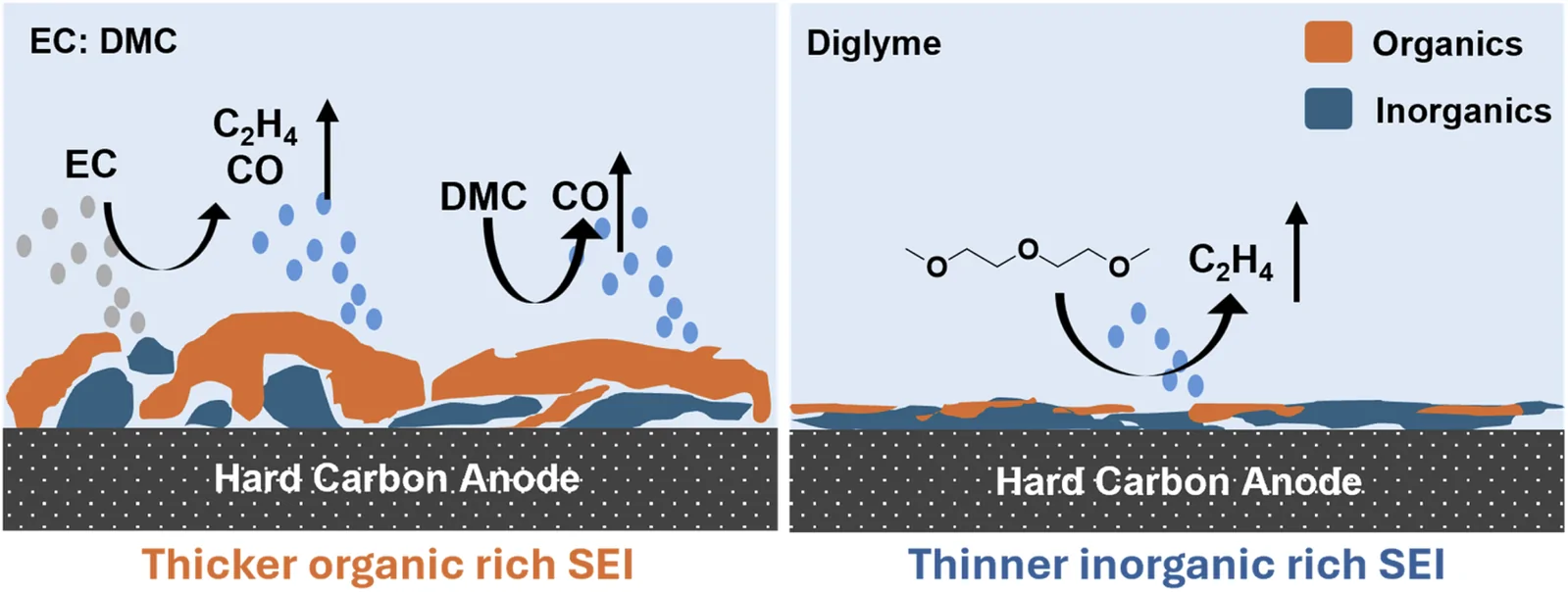

## 1. Introduction

Sodium-ion batteries (SIBs) are being increasingly explored as a sustainable electrochemical energy storage technology that complements lithium-ion batteries (LIBs). SIBs are particularly attractive for applications such as short-range electric mobility and grid-scale energy storage.^1^ This positioning arises from inherent electrochemical limitations, as SIBs typically exhibit lower energy density than LIBs, primarily due to the lower redox potential of sodium compared to lithium (Na^+^/Na ≈ −2.71 V *vs.* SHE, compared to Li^+^/Li ≈ −3.02 V *vs.* SHE).^2^ However, SIBs offer significant advantages in terms of elemental abundance, cost, and long-term sustainability, thereby providing a pathway to mitigate challenges associated with lithium technologies, including diminishing resource availability and geopolitical constraints on lithium supply chains.^3^ Although substantial insight can be transferred from LIB research to SIBs, materials and mechanisms are not always directly analogous, and electrode chemistries that perform well in LIBs often exhibit markedly different behaviour in Na-based systems. For example, graphite, which readily intercalates Li^+^ in LIBs but is ineffective for Na^+^ storage. This limitation is attributed to the thermodynamic constraints faced by sodium to form stable binary graphite intercalation compounds (b-GICs), whereas Li and K form LiC_6_ and KC_8_, respectively. The most stable Na-graphite compound reported is NaC_64_, rendering Na intercalation into graphite thermodynamically unfavourable.^4–6^ Consequently, hard carbon has been adopted as the state-of-the-art anode material for SIBs. Where studies show that graphite exhibit limited capacity largely due to plating and stripping of Na at the interface, hard carbons have been reported to exhibit high discharge capacities of ∼300–320 mAh g^−1^ depending on the microstructure.^6,7^ Despite its promise, hard carbon anodes remain challenged by limited long-term cycling stability, issues that are closely linked to interfacial reactions and solid electrolyte interphase formation.^8^

The solid electrolyte interphase (SEI) is an electronically insulating and ionically conductive layer formed at the anode–electrolyte interface as a result of electrolyte reduction during the initial formation cycles, and its chemical stability and mechanical integrity are therefore critical determinants of long-term cycling performance.^8^ In Na-ion batteries, a key challenge arises from the comparatively higher solubility of sodium-based SEI components relative to their lithium analogues, which promotes partial dissolution of the interphase into the electrolyte.^9^ This dissolution exposes fresh electrode surface, triggering renewed electrolyte decomposition and repeated SEI reformation. Such a dynamic and non-passivating interphase leads to continuous consumption of sodium inventory and electrolyte, resulting in progressive capacity loss and degraded cycle life.^8^ SEI formation is governed by multiple factors, both physical and chemical factors, among which the electrolyte solvent plays a vital role, as it mediates ion transport through solvation and largely dictates the primary reduction reactions that define SEI composition, morphology, and stability.^10^ Accordingly, a range of solvent systems has been explored in SIB research, including organic, ionic liquids, and water-in-salt electrolytes.^11^ Among these, ether-based organic electrolytes have garnered particular attention due to their lower Na^+^ desolvation energies and faster interfacial kinetics, which are associated with improved electrochemical performance.^12^ As reported by Lv *et al.*, in ether-based electrolytes, Na^+^ typically inserts while retaining one coordinating solvent molecule, such that the effective kinetic barrier corresponds to the second-to-last desolvation step (≈88 kJ mol^−1^ for DME and ≈ 100 kJ mol^−1^ for DEGDME), whereas in carbonate-based electrolytes Na^+^ inserts predominantly in a fully desolvated state and the last desolvation step therefore constitutes the dominant barrier (≈109 kJ mol^−1^ for EC and ≈ 128 kJ mol^−1^ for DEC).^13^ In parallel, the higher LUMO energies of glymes relative to carbonate solvents confer greater reductive stability, leading to reduced solvent decomposition and the formation of thinner, more stable, and ionically conductive SEI layers compared with those typically formed in carbonate-based electrolytes.^14^

As of the current status of the literature, SEI chemistry is still predominantly inferred from post-mortem and *ex situ* characterization techniques, which provide valuable but inherently static information and cannot resolve transient electrolyte decomposition pathways occurring during interphase formation.^15,16^ As a result, the mechanistic origin of solvent-specific degradation reactions and their direct connection to SEI growth dynamics remain insufficiently understood. In this regard, *operando* gas-evolution detection techniques provide a powerful means to probe electrolyte degradation processes.^17,18^ Because the reductive decomposition of electrolyte components associated with SEI formation produces gaseous by-products, the identity and temporal evolution of these gases can be correlated with specific solvent- or salt-derived breakdown pathways. Such measurements therefore enable mechanistic attribution of electrolyte decomposition reactions and provide insight into the chemical composition and growth dynamics of the SEI. Several *operando* gas-analysis techniques, including differential electrochemical mass spectrometry (DEMS) and on-line electrochemical mass spectrometry (OEMS), have been employed to study gas evolution in battery systems.^17,19^ While these methods yield valuable information on degradation processes, their sensitivity and quantitative reliability can be limited by non-uniform electrochemical configurations and variations in cell design. In contrast, chip-based electrochemistry-mass spectrometry (EC-MS) offers a standardized cell architecture and high detection sensitivity, reaching the picomole-per-second regime, thereby enabling the resolution of subtle gas-evolution events during electrochemical operation.^20,21^ In the present work, EC-MS is employed to directly compare solvent-dependent gas evolution and electrolyte decomposition pathways in Na-ion cells using hard carbon anodes and two electrolyte systems, a 1 : 1 mixture of EC : DMC and diglyme, under identical electrochemical conditions. This approach provides mechanistic insight into how solvent chemistry governs interfacial degradation reactions and the resulting SEI composition and stability.

## 2. Experimental

### 2.1. Hydrothermal carbonisation of hard carbons

Hydrothermal carbonization (HTC) was employed as the synthesis route due to its superior control over hard-carbon morphology compared with direct carbonization, as HTC-derived hard carbons are intrinsically spherical, which enhances ion transport and promotes uniform and stable solid electrolyte interphase (SEI) formation.^22^ In this study, hard carbons were synthesized by preparing an aqueous solution containing 10 wt% d-glucose (Sigma-Aldrich) in deionized water which was transferred into a 125 mL Teflon-lined stainless-steel autoclave (Parr Instruments, USA), sealed, and heated in a convection oven (Memmert GmBH, Germany) at 230 °C for 12 h. After natural cooling to room temperature, the resulting hydrochar was collected by filtration and dried at 80 °C for 48 h. The dried hydrochar was subsequently carbonized in a tube furnace (Carbolite Gero GmBH Germany) at 1500 °C for 2 h under continuous nitrogen flow and a heating rate of 5 °C min^−1^, after which the obtained hard carbon, hereafter named as G1500, was mechanically ground and used for further characterization and electrochemical evaluation.

### 2.2. Electrode preparation for half-cell setup

The synthesized hard carbon, hereafter denoted as G1500, was mixed with carboxymethyl cellulose (CMC) binder in a mass ratio of 9 : 1 (HC : CMC) and cast onto an aluminium foil current collector with a wet thickness of approximately 200 µm. The coated electrodes were cut into 8 mm × 8 mm squares and dried under vacuum at 80 °C overnight. The dried electrodes were subsequently transferred into an argon-filled glovebox (H_2_O < 0.5 ppm, O_2_ < 0.5 ppm, MBraun GmbH, Germany), where CR2032-type half-cells were assembled using sodium metal discs as the counter electrode (14 mm diameter, 1 mm thickness, Canrd Technology Co., Ltd, China) and GF/A glass fibre separators (Whatman, 16 mm diameter). Two electrolytes were investigated: 1 M NaPF_6_ in (1 : 1) ethylene carbonate/dimethyl carbonate (EC:DMC, Elyte GmbH, Germany) and 1 M NaPF_6_ in diglyme (Canrd Technology Co., Ltd, China). The EC:DMC solvent mixture was dried using molecular sieves prior to use, whereas the diglyme electrolyte was used as received. The water content of both electrolytes was determined using Karl Fischer coulometric titration (917 Coulometer, Metrohm AG, Switzerland) and was found to be 10.1 ppm and 23.33 ppm for EC:DMC and diglyme based electrolytes, respectively. The assembled cells were subjected to rate capability testing using a Landt battery cycler, followed by long-term cycling for 100 cycles at a current rate of 0.5 C, at ambient room temperature.

### 2.3. *Operando* electrochemistry mass spectrometry

#### 2.3.1. Electrode preparation for EC-MS

The preparation of electrodes for EC-MS measurements differed from the conventional electrode casting procedure described above and was specifically adapted to meet the requirements of *operando* gas analysis. Instead of being cast onto aluminium foil, the EC-MS electrodes were fabricated by spray coating. In this process, G1500 was mixed with CMC binder in a mass ratio of 9 : 1 and subsequently diluted with deionized water to form a homogeneous sprayable ink. The ink was uniformly sprayed using a hand-held spray coater onto an aluminium mesh current collector (EL-Cell GmBH, Germany) placed on a hot plate maintained at 80 °C to promote rapid solvent evaporation and ensure stable film formation. After drying, the coated mesh was punched into 6 mm diameter circular electrodes, further dried under vacuum at 80 °C, and then used for subsequent electrochemical testing. For counter electrodes, 5 mm diameter sodium metal electrodes were cut from the 14 mm sodium chips used in half-cell experiments, thereby maintaining identical sodium thickness across all measurements, and 20 µL of electrolyte was used for each cell configuration. The assembled EC-MS cells were then subjected to galvanostatic cycling at 0.1 C to enable time-resolved *operando* gas evolution analysis.

#### 2.3.2. EC-MS cell assembly

*Operando* gas evolution measurements were performed using a non-aqueous Electrochemistry-Mass Spectrometry (EC-MS) membrane chip (SpectroInlets ApS, Denmark) mounted onto the interface block of the EC-MS system and was subsequently the chip was pumped down. All cell components were assembled and sealed inside an argon-filled glovebox (H_2_O < 0.1 ppm, O_2_ < 0.1 ppm, MBraun GmbH, Germany). The non-aqueous EC-MS cell (schematically shown in Fig. 1) was assembled by first placing a sodium metal chip (5 mm diameter) as the counter electrode onto the stainless-steel plunger. A glass microfibre separator (Whatman GF/A, 5 mm diameter) was then placed on top of the sodium metal electrode, onto which 10 µL of electrolyte was added to ensure complete wetting. The working electrode, consisting of a 6 mm diameter aluminium mesh coated with G1500, was subsequently placed above the separator, followed by the addition of a further 10 µL of electrolyte to the electrode stack. A Celgard separator (7 mm diameter) was placed between the working electrode and the microporous membrane chip to prevent mechanical damage to the membrane from the aluminium mesh and to ensure stable gas sampling. A schematic of the EC-MS setup is shown in Fig. 1. The assembled cell was mounted onto the EC-MS interface block, ensuring electrical contact between the stainless-steel contact and the working electrode, and then fully sealed. The contacts of the assembled cell were then further connected to a potentiostat (Biologic SP-200). After a rest period of 1.5 h, the cell was cycled for two formation cycles at a rate of 0.1 C to monitor gas evolution. Mass-to-charge ratios (*m*/*z*) from 2 to 108 were continuously detected during the experiment.

**Fig. 1:**
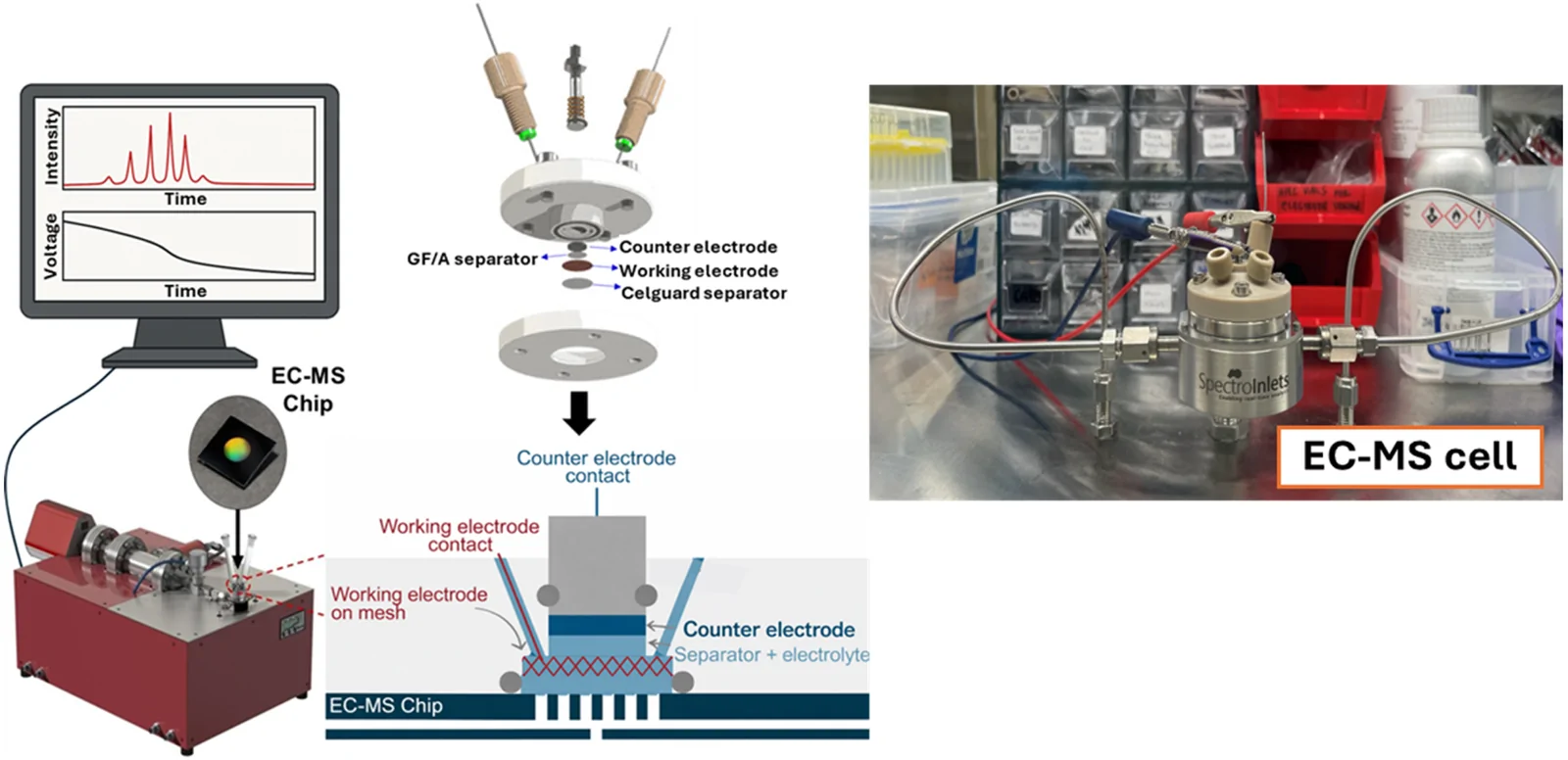
Schematic illustration of the chip-based electrochemistry-mass spectrometry (EC-MS) setup used for gas evolution measurements. Adapted from Thornton *et al.*, 2024. Ref. 23.

#### 2.3.3. Data interpretation

The electrochemical and mass spectrometric data were processed using the *ixdat* (v 0.3.0) python package.^24^ Raw ion-current signals were synchronized with the corresponding electrochemical data acquired from the potentiostat. Gas signals were quantified using experimentally determined calibration factors obtained by calibrating the MS using calibration gas. Baseline correction was applied manually to remove background contributions and instrumental drift, enabling extraction of net gas evolution profiles as a function of time and cell potential. The quantified gas evolution rates were subsequently correlated with electrochemical features to facilitate mechanistic interpretation of electrolyte decomposition processes.

## 3. Results and discussion

### 3.1. Electrochemical performance

The rate capability and long-term cycling performance of G1500 in 1 : 1 EC:DMC and diglyme electrolytes are shown in Fig. 2(a) and (b), respectively. The rate capability results show that half-cells using diglyme perform significantly better than those using EC:DMC. At 0.1 C, both electrolyte systems deliver similar reversible capacities of about 305 mAh g^−1^ and initial coulombic efficiencies (ICE) of 91.2 ± 0.8% and 93.82 ± 0.5% for EC:DMC and diglyme based electrolytes, respectively. However, as the C-rate increases, the performance difference becomes pronounced. Cells employing EC:DMC exhibit diminished capacity, indicating higher internal resistance, whereas diglyme-based cells retain much higher capacities. At 2 C, the discharge capacity in diglyme remains around ∼270 mAh g^−1^, while the EC:DMC system drops to approximately 50 mAh g^−1^. This behaviour can be explained by the lower Na^+^ solvation and desolvation energy in diglyme compared with EC:DMC, which enables faster charge-transfer kinetics and superior high-rate performance.^13^ Also, from Fig. 2(b), it could be observed that the cells employing diglyme as the solvent exhibit a higher capacity and stability over 100 cycles compared to the cells employing EC:DMC.

**Fig. 2:**
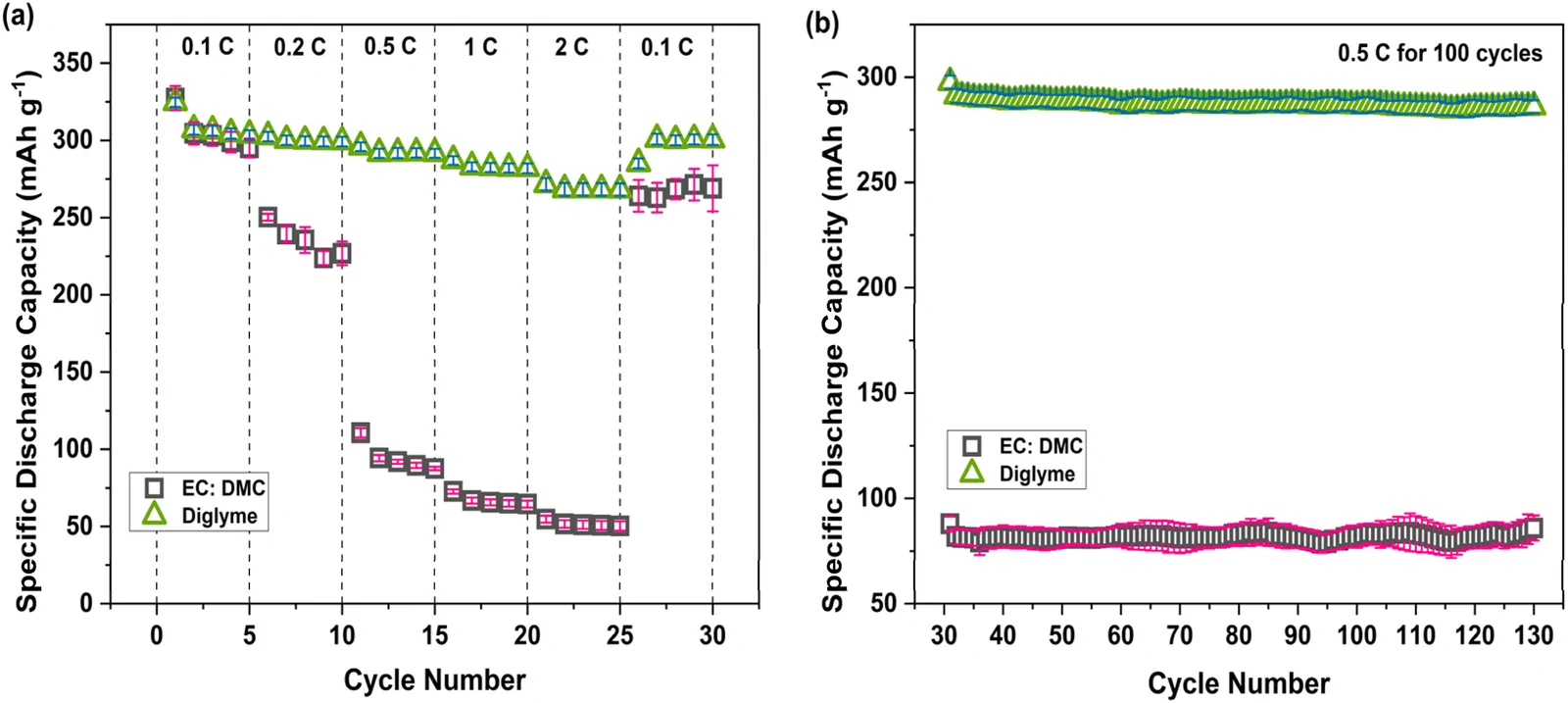
(a) Rate capability and (b) cycling stability over 100 cycles of G1500 hard-carbon electrodes in Na half-cells using 1 M NaPF_6_ in EC:DMC (1 : 1) and diglyme electrolytes. Cells were cycled galvanostatically within a voltage window of 0.001–2.5 V *vs.* Na^+^/Na at ambient temperature, and capacities are reported based on the mass of active material.

### 3.2. Electrochemistry mass spectrometry analysis

The cycling profiles of the EC-MS cell and the conventional coin cell for both electrolyte systems are shown in Fig. 3. The EC-MS cell exhibits electrochemical behaviour comparable to that of the coin cell, with only a minor loss in capacity, which is attributed to partial electrolyte drying under *operando* conditions. This capacity loss is not systematic and can be mitigated through minor modifications to the cell configuration. Importantly, the voltage profile retains the same characteristic shape as that of the coin cell, including the first sloping region (first discharge curve) above 0.1 V associated with the onset of electrolyte decomposition, which remains identical and highly reproducible across measurements.

**Fig. 3:**
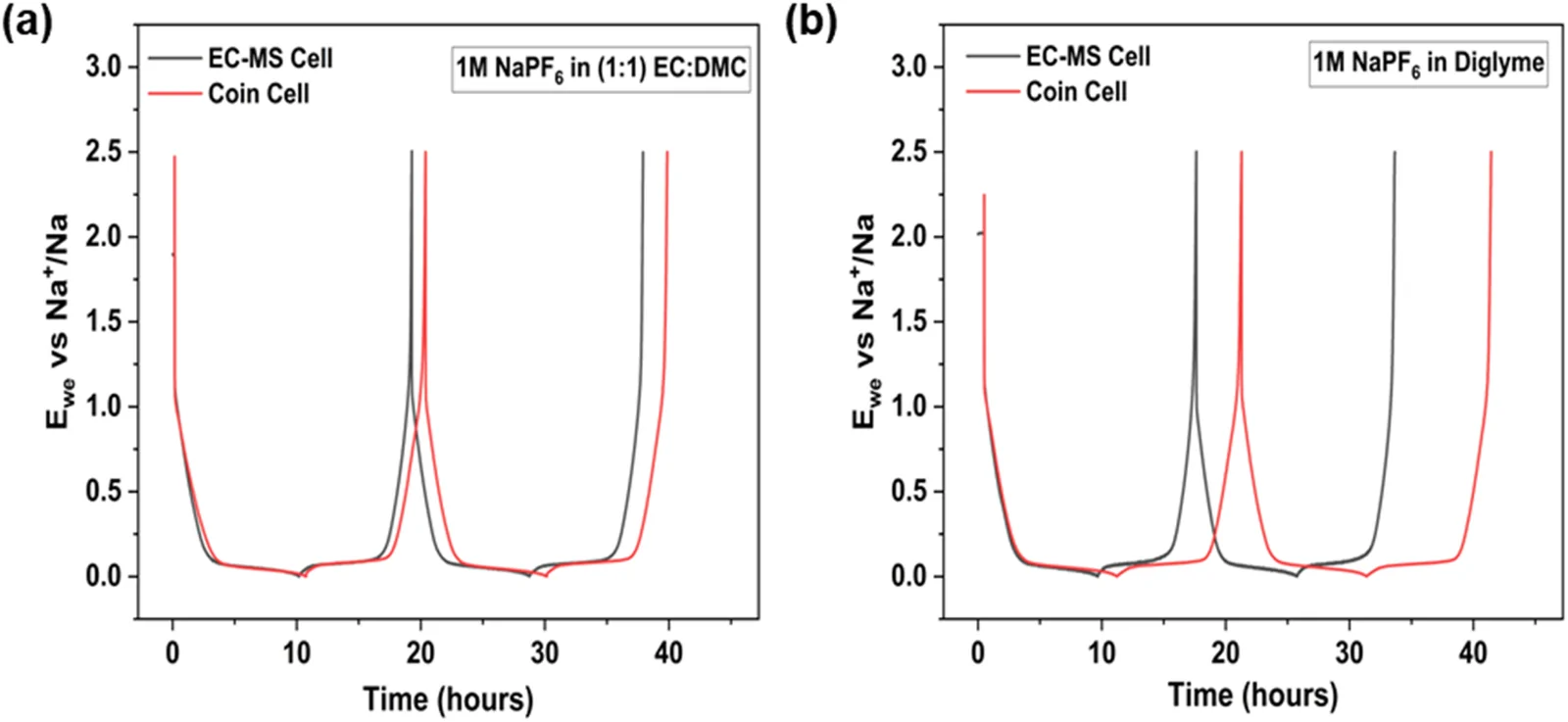
Comparison of galvanostatic cycling behaviour between the *operando* chip-based EC-MS cell and a conventional CR2032 Na half-cell using 1 M NaPF_6_ in (a) EC:DMC (1 : 1) and (b) diglyme. Cells were cycled within a voltage window of 0.001–2.5 V *vs.* Na^+^/Na at 0.1 C under ambient conditions.

The *operando* gas-evolution profiles for the two solvent systems are shown in Fig. 4(a) and (b), respectively. For cells operated with EC:DMC electrolyte (Fig. 4(a)), ethylene (C_2_H_4_) is the dominant gaseous product, arising from the reductive decomposition of EC with concomitant formation of sodium ethylene dicarbonate (SEDC) and Na_2_CO_3_, as described by the reaction pathways in eqn (1) and (2). This assignment is consistent with reported XPS and surface-sensitive spectroscopic studies on hard-carbon anodes in Na-ion systems, which identify SEDC and Na_2_CO_3_ as the principal SEI constituents when EC is employed as the solvent.^16,25^ By analogy with Li-based systems, if there is any breakdown of SEI, SEDC might also undergo secondary reduction to yield C_2_H_4_ and Na_2_CO_3_ (eqn (3)).^26^1
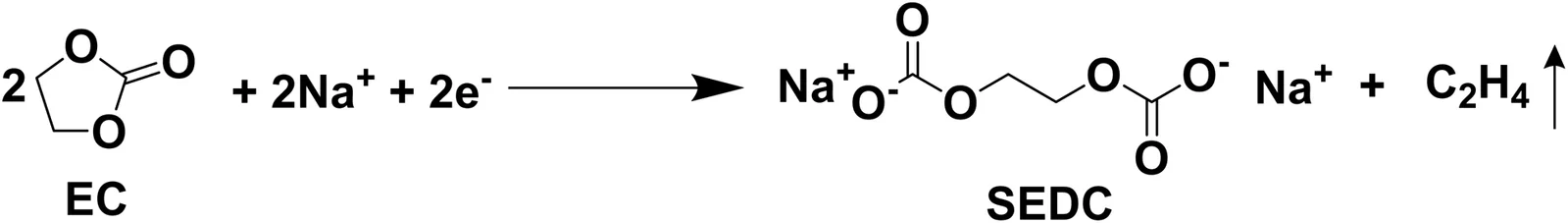
2
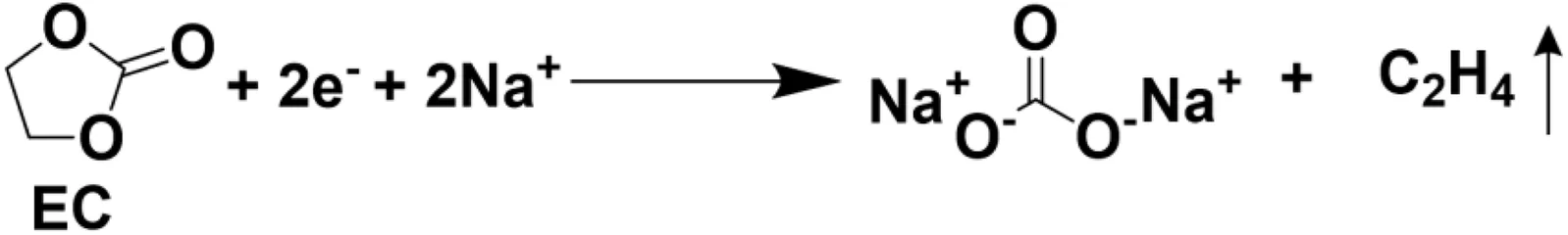
3
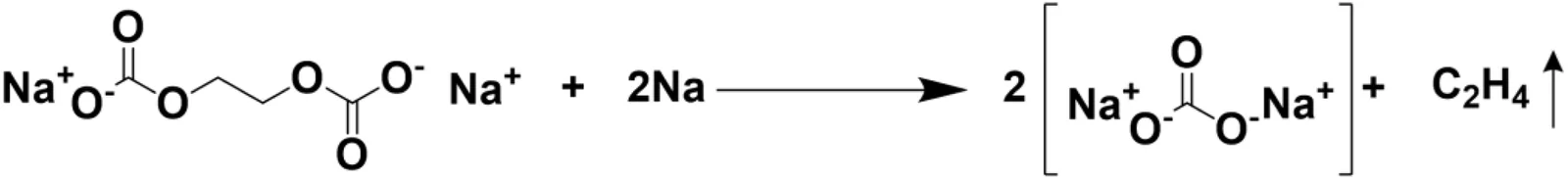


**Fig. 4:**
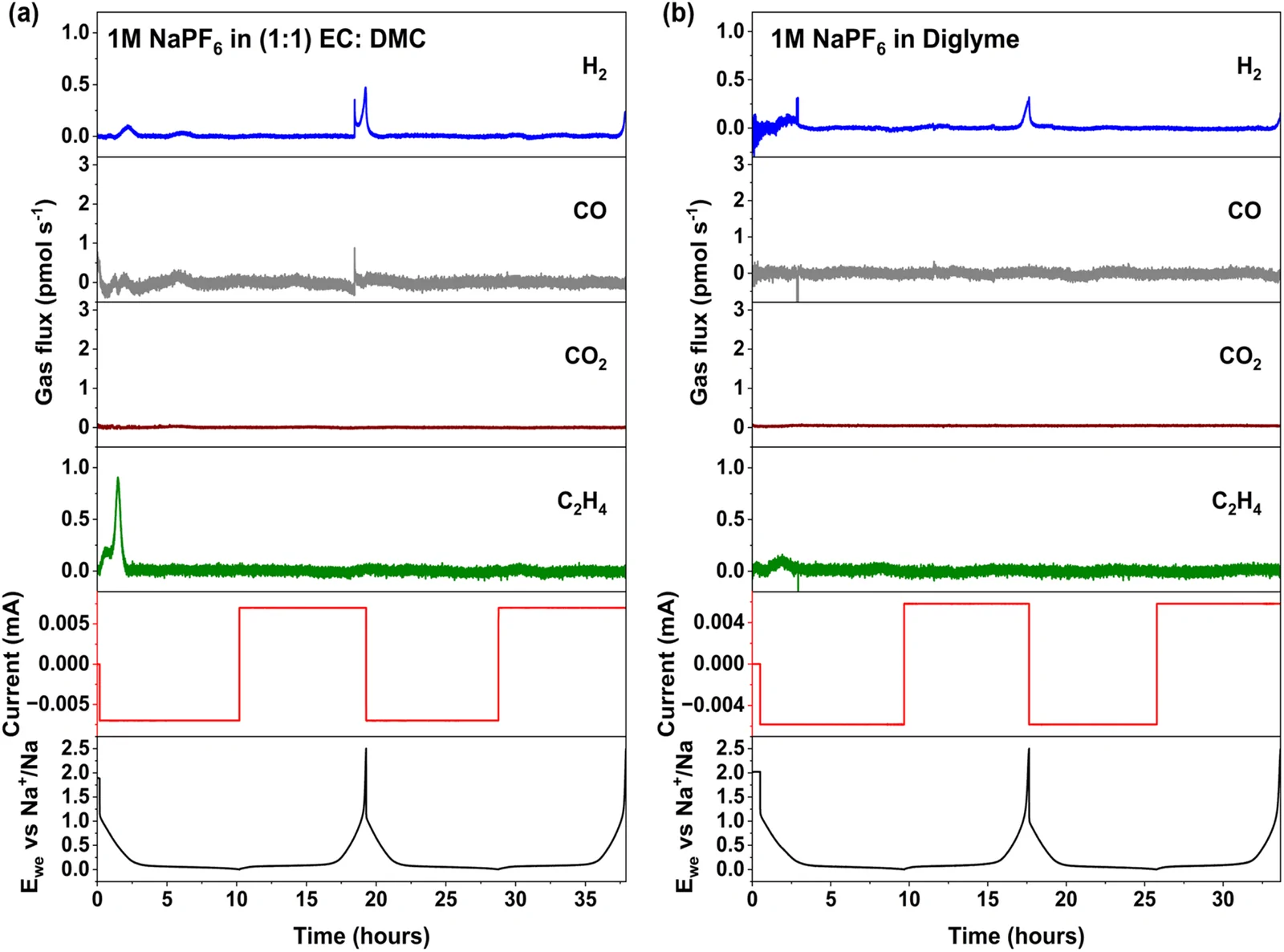
Gas evolution profiles from on-chip electrochemistry-mass spectrometry of G1500 hard carbon *vs.* Na half-cells employing 1 M NaPF_6_ in (a) (1 : 1) EC:DMC and (b) diglyme electrolytes. Cells were cycled galvanostatically within a voltage window of 0.001–2.5 V *vs.* Na^+^/Na at a rate of 0.1 C under ambient conditions.

In contrast, only trace amounts of CO are detected, indicating the minimal reductive decomposition of DMC, which is commonly associated with CO formation (eqn (4)).^27^ CO formation may also arise from a minor EC reduction pathway, as proposed by Onuki *et al.* (eqn (5)); however, this route is considered secondary to the dominant EC decomposition mechanism leading to SEDC and Na_2_CO_3_.^28^ No CO_2_ evolution is observed throughout cycling, implying that the carbonate species formed do not undergo further oxidation to CO_2_ within the operative potential window.^26^ Moreover, CO_2_ generation from decomposition of carbonate species has been reported to be catalysed by HF impurities; the absence of a CO_2_ signal therefore suggests negligible impurity-driven CO_2_ formation pathways.^29^4
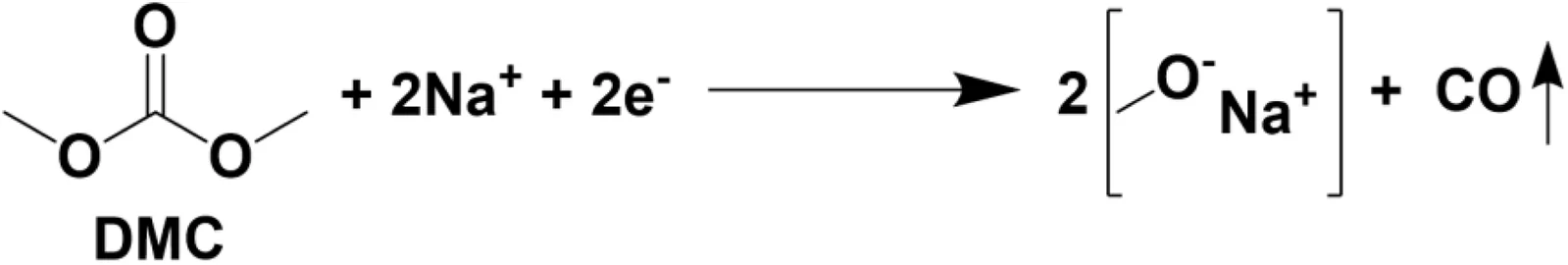
5
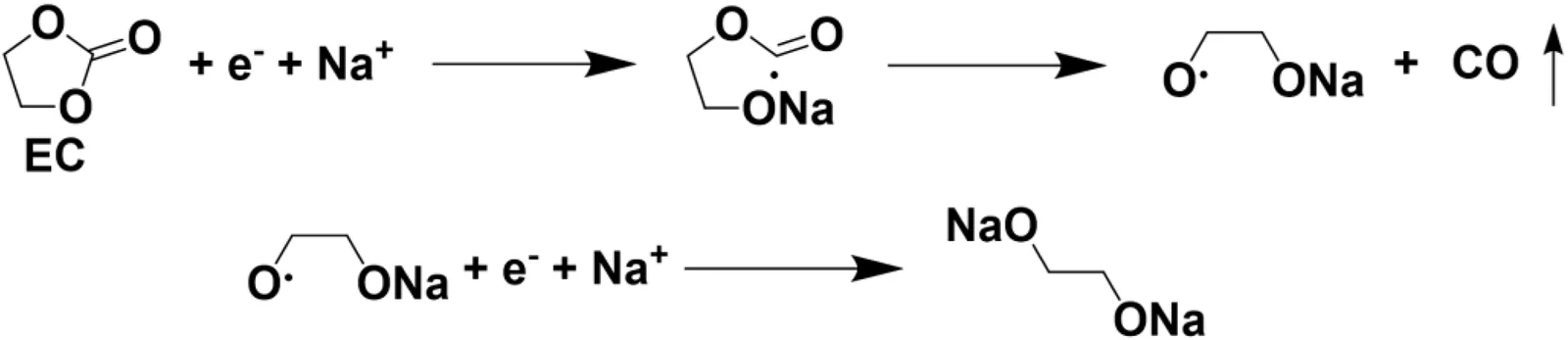


A small, broad H_2_ signal is observed during the initial sloping region, followed by a sharper peak at approximately 2.5 V. Hydrogen evolution is commonly attributed to the electrochemical reduction of residual water present in the cell (eqn (6)).^30^ An additional contribution may arise from the reduction of protic impurities such as alcohols or organic acids (eqn (7) and (8)), with HF being a typical by-product of NaPF_6_ hydrolysis.^31,32^ Because several reactions can contribute to H_2_ formation, a single dominant pathway cannot be assigned with certainty and needs further investigation. The sharp H_2_ peak on discharge at 2.5 V may originate from reactions at the Na metal counter electrode, where highly reducing conditions (∼0 V *vs.* Na/Na^+^) might promote the conversion of trace protic species to H_2_, and its origin therefore requires further investigation.

6 H_2_O + e^−^ → OH^−^ + 1/2H_2_↑

7 NaOH + Na^+^ + e^−^ → Na_2_O + 1/2H_2_↑

8 HF + Na^+^ + e^−^ → NaF + 1/2H_2_↑

Collectively, these observations indicate that SEI formation when employing a (1 : 1) EC:DMC electrolyte is primarily governed by EC decomposition, yielding SEDC and Na_2_CO_3_ as the dominant interphase components. One of the plausible reasons underlying this behaviour is the preferential solvation of Na^+^ by EC over DMC in the bulk electrolyte, resulting in an EC-rich primary solvation shell.^33^ The Na-EC solvate migrates toward the negatively polarised electrode during sodiation. At the interface, the solvated Na^+^ accumulates in the inner Helmholtz plane (IHP), where partial desolvation and initial electron transfer take place. This solvation-driven enrichment of EC at the IHP promotes the reductive reactions toward EC, promoting its ring-opening decomposition and dictating the chemical nature of the nascent SEI. This suggests that the SEI formed in carbonate-based electrolytes is predominantly organic in nature.

The gas evolution profile for 1 M NaPF_6_ in diglyme is shown in Fig. 4(b). In contrast to EC:DMC, minimal gas evolution is observed, indicating the higher reductive stability of diglyme, consistent with its higher LUMO relative to carbonate solvents. This behaviour implies a substantially lower degree of electrolyte decomposition and the formation of a thinner and more stable SEI. C_2_H_4_ evolution is detected during the first discharge, corresponding to a minor but measurable contribution to the overall irreversible capacity; although reductive decomposition pathways for diglyme in Na-based systems have not yet been clearly established, cleavage of ether backbone bonds under strongly reducing conditions may account for the observed ethylene evolution and requires further investigation.^34^ The limited gas evolution suggests the formation of a predominantly inorganic SEI in diglyme-based electrolytes. Similar H_2_ signals are also observed, and their origin can be attributed to the same processes described for the EC:DMC above. In both electrolyte systems, gas evolution is observed exclusively during the first discharge cycle. This suggests that, despite the dynamic nature of the SEI and its continued evolution during cycling, the primary formation of the SEI occurs within this initial reductive window. A summary of the gaseous species detected, and comparison of total gas evolved during the first cycle for both solvent systems is presented in Table 1 and Fig. 5, respectively. Based on the discussion above, the information obtained from the gas analysis can provide an indication of the SEI composition based on the identity and quantity of gases evolved, as well as a rough estimate of the amount of SEI formed. From this, a possible representation of the SEI structure can be postulated and subsequently verified using complementary analytical imaging techniques. Based on these observations, a schematic representation of the proposed SEI structures for both electrolyte systems is shown in Fig. 6, where the SEI formed in EC:DMC (carbonate) electrolytes is comparatively thicker, while that formed in diglyme (ether) electrolytes is thinner, consistent with trends reported in literature.^35^

**Table 1:** Summary of evolved gaseous species detected by ECMS and proposed reduction pathways

Gases observed		Solvent systems			
Species	*m*/*z*	EC:DMC	Possible SEI species	Diglyme	Possible SEI species
H_2_	2	Yes; H_2_O or H^+^ species reduction	Oxides and fluorides	Yes; H_2_O or H^+^ species reduction	Oxides and fluorides
CO	28	Yes; low amounts; possible DMC and EC decomposition	Sodium methoxide	Not detected	
CO_2_	44	Not detected		Not detected	
C_2_H_4_	26, 27, 28	Yes; EC decomposition	Sodium ethylene dicarbonate, Na_2_CO_3_	Yes; low amounts; possible diglyme backbone scission	Possibly organics

**Fig. 5:**
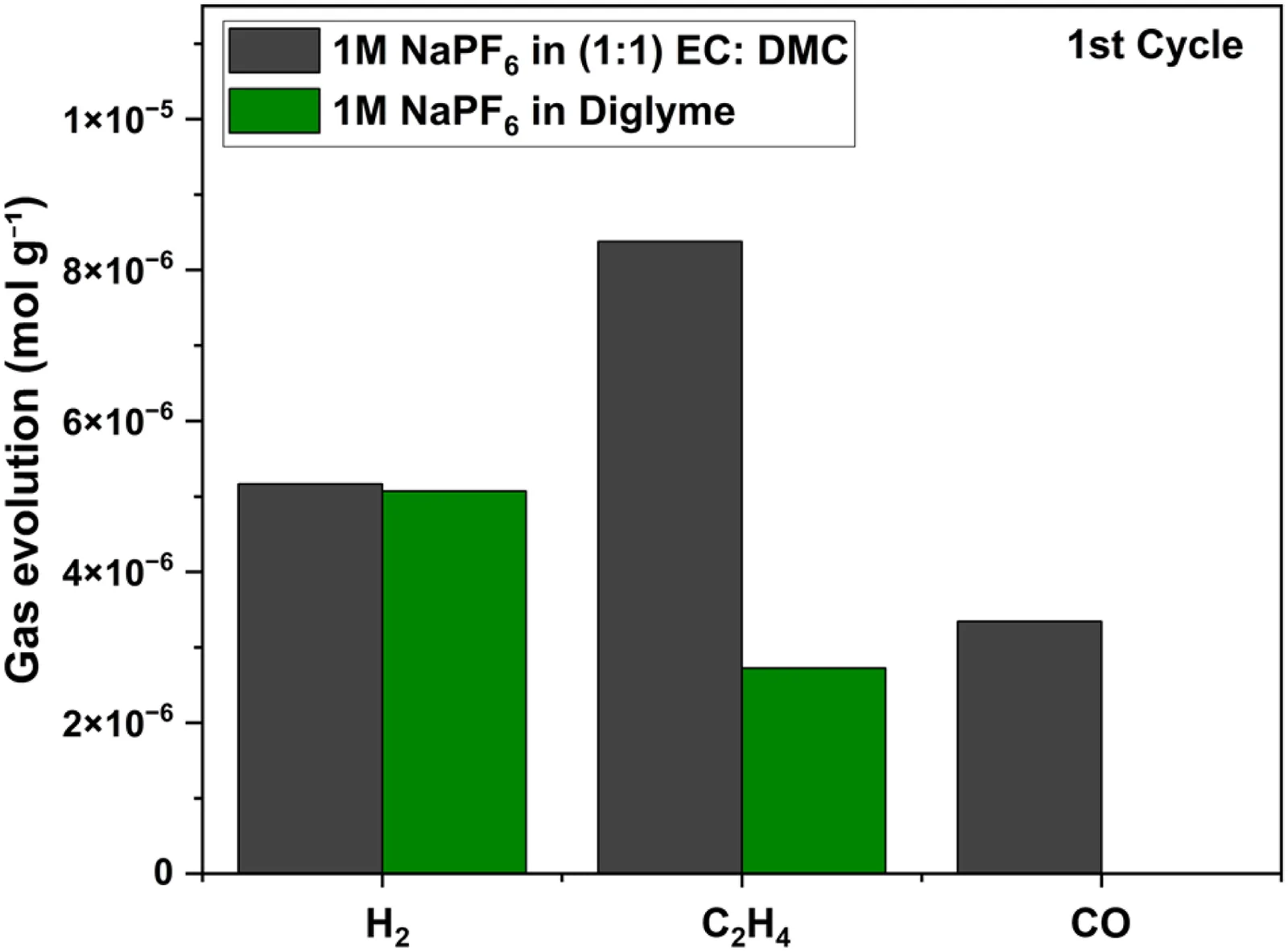
Comparison of total gas evolution (1st cycle) of 1 M NaPF_6_ in (1 : 1) EC:DMC and diglyme, normalized to active material mass.

**Fig. 6:**
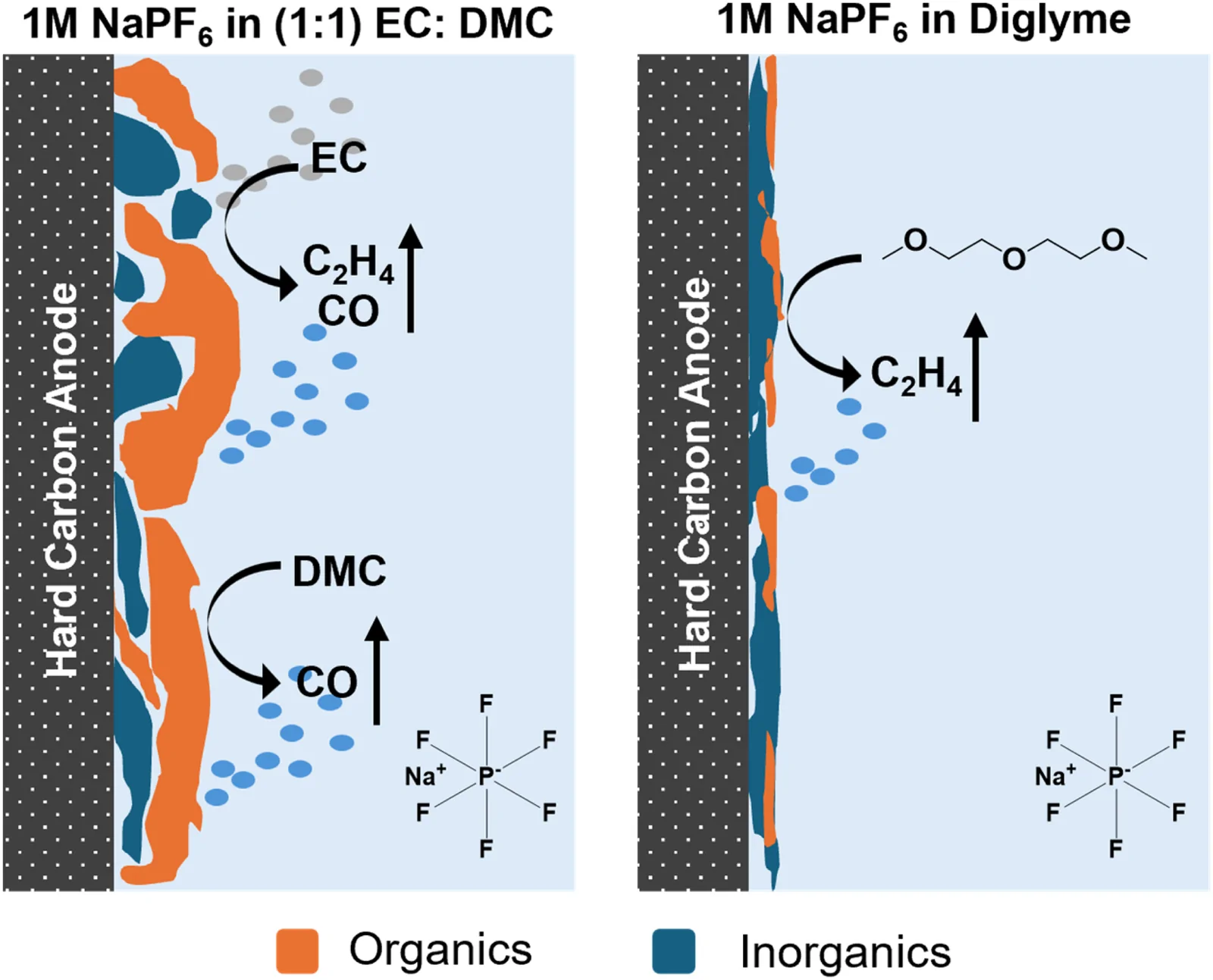
Schematic representation of the proposed SEI structures formed in carbonate-based and glyme-based electrolytes.

## 4. Conclusion

In conclusion, this study demonstrates that solvent selection exerts a pivotal influence on electrolyte decomposition pathways and interphase formation in sodium-ion batteries, as resolved by *operando* electrochemistry-mass spectrometry. When a (1 : 1) EC:DMC electrolyte is employed, ethylene (C_2_H_4_) is the dominant gaseous product, while only trace amounts of CO and no detectable CO_2_ are observed. This behaviour indicates that SEI formation is governed primarily by EC reduction, leading to the formation of SEDC and Na_2_CO_3_. In contrast, the use of diglyme leads to a significant suppression of gas evolution, with only minute quantities of C_2_H_4_ detected, reflecting the higher reductive stability of ether-based electrolytes and a reduced extent of solvent decomposition. Although, H_2_ has been observed in both the systems, the origin is not exactly known and requires further investigation. Electrolyte evaporation of volatile solvents remains a current limitation of the EC-MS cell configuration, as observed by the cycling comparison between conventional coin cells and *operando* on-chip EC-MS cells, where capacity fading is observed in the latter. Nevertheless, the electrochemical behaviour remains reproducible, with similar cycling profiles and a comparable first-discharge profile, during which the majority of gas evolution occurs, indicating that the key interfacial processes are captured by the setup. Overall, the EC-MS results provide direct, time-resolved evidence of solvent-dependent differences in SEI chemistry, establishing a clear link between possible electrolyte decomposition pathways and interphase formation, and offering a mechanistic framework that can be further validated using complementary advanced characterisation techniques.

## Conflicts of interest

There are no conflicts to declare.

## Data Availability

Data will be available upon request to the authors.
